# The state of the art and future of climate risk insurance modeling

**DOI:** 10.1111/nyas.15255

**Published:** 2024-11-12

**Authors:** Michiel W. Ingels, W. J. Wouter Botzen, Jeroen C. J. H. Aerts, Jan Brusselaers, Max Tesselaar

**Affiliations:** ^1^ Institute for Environmental Studies (IVM) Vrije Universiteit Amsterdam Amsterdam The Netherlands; ^2^ Department of Flood Risk Management Deltares Delft The Netherlands

**Keywords:** actuarial models, catastrophe models, climate change, insurance, natural disaster risk

## Abstract

This study provides a comprehensive review of the literature on climate risk insurance modeling to identify lessons learned and knowledge gaps to be addressed by future research. These models are increasingly relevant due to the rising losses attributable to climate change. Insurance models estimate risk for different perils and simulate risk‐related parameters for insurance schemes, such as premiums and deductibles. Most forward‐looking models indicate that climate change and socioeconomic developments highly exacerbate future risk and increase insurance premiums. Various studies recommend charging risk‐based premiums to incentivize adaptation efforts that limit this increase in climate risks. Other findings point toward introducing public–private insurance to cope with climate change and enhance risk spreading by introducing insurance purchase requirements or insurance products that cover multiple climate risks. Gaps that we identify in this literature include an underrepresentation of insurance assessments for developing countries and for hazards other than flooding. Additionally, we note a lack of research into insurance for non‐agricultural commercial sectors. Furthermore, less than half of the studies take a forward‐looking approach by incorporating climate change scenarios, and an even smaller percentage consider socioeconomic development scenarios. This limitation shows that current methods require additional development for assessing the effects of future climate risk on insurance. We recommend that future research develops such forward‐looking models, considers using a more refined spatial scale, broadens geographical and hazard coverage, and includes the commercial sector.

## INTRODUCTION

Global warming will increase the frequency and severity of natural disasters.^1^ Future risk will increase due to trends in climate extremes and socioeconomic developments like urbanization and population growth. The number of natural disasters with high economic impacts has tripled since the 1980s, and this trend is expected to continue into the future.^2^ With an increasing number of individuals residing in hazard‐prone areas, the potential for losses from climate‐related events is anticipated to rise.^1^ Natural disasters and future climate risk lead to significant direct and indirect damage for society.^3^ Insurance can be a tool to soften this burden on society by compensating the losses to households and private businesses.^4^ An efficiently working insurance system accelerates recovery after a natural disaster, minimizes the damage to the economy, and can improve the resilience of communities against natural disasters by incentivizing risk reduction.^5^ However, as of now, less than half of the global natural disaster losses are covered by insurance.^6^

Designing an effective insurance system to cover losses from natural disasters is a complex task.^7^ A viable insurance system for natural disasters uses a multitude of variables to optimize its operations, including the spatial and temporal pooling of risk, the diversification of underwritten risk with other types of risks, and premium‐setting rules. In addition, the increase in natural hazards due to climate change^1^ and the increase in the exposure of assets and people^2^ necessitate larger (future) capital requirements for insurers. Consequently, this results in higher premiums for consumers, diminishing the appeal of purchasing insurance.^5^ Other challenges for developing a viable insurance system are the (often unexpected) high impacts of catastrophic events.^8^ Furthermore, climate change is often not addressed in current insurance schemes,^9^ and there is much uncertainty in future climate risk projections, which increases uncertainty in future premium settings.^5^, ^10^

The modeling of climate‐related risk insurance is an emerging research field to prepare the insurance sector for the increasing natural disaster risk. By assessing how climate change may stress the insurance sector, strategies can be developed to enhance the resilience of this sector to increasing climatic risks. For example, insurance could stimulate risk‐conscious decision‐making by policyholders, which may limit the impact of future climatic hazards. In light of the necessity for policyholders to make decisions considerably in advance of climate change impacts, it is imperative that the design of insurance policies embraces a long‐term future‐oriented outlook.

A key foundation of a climate risk insurance model is accurately estimating current and future risk through catastrophe modeling, actuarial approaches, or probability/theoretical methods. Over the last 20 years, numerous climate hazard and risk models for different perils have been developed.^11^, ^12^, ^13^, ^14^, ^15^, ^16^, ^17^

In addition, a climate risk insurance model can be applied to assess the impacts of climatic risks on how supply and demand for insurance develop over time and space. A commonly employed model type for this purpose is an insurance supply model, which concerns the pricing of insurance contracts by simulating (risk‐based) premiums (e.g., Aerts and Botzen^18^). On the demand side, partial equilibrium models aim to simulate supply and demand in an insurance market or consider the effect of insurance on equilibrium conditions between marginal cost and marginal revenue for a business. In this way, it is possible to derive insights about insurance uptake (e.g., Tesselaar et al.^19^) or how insurance can incentivize adaptation (e.g., Hudson et al.^20^). Recently, agent‐based insurance models have been developed, which aim to simulate the complex interactions in an insurance market among individual autonomous consumers, insurers, and the government.^21^

Although the recent research has reviewed climate insurance studies in a broad context (including sustainability issues),^22^ there is no systematic review of climate risk models for the insurance sector. Therefore, this paper primarily aims to review and synthesize the current literature about climate risk models for the insurance sector. This process will identify the key building blocks of such models, best practices, and lessons learned and provide recommendations for future model development. Because existing models are already used by the European Insurance and Occupational Pensions Authority^19^, ^23^ or the European Central Bank (ECB),^24^ our review will offer valuable insights to policymakers and the insurance sector about how to address future climate challenges.

The remainder of this paper is organized as follows: The Methods section describes how the review has been conducted. The Results section reviews the literature by summarizing our findings in three parts: general model types, the risk component, and the insurance model component. The Discussion section discusses the main research challenges and provides recommendations for future research. The Conclusion section concludes the paper.

## METHODS

### Paper selection

**FIGURE 1:**
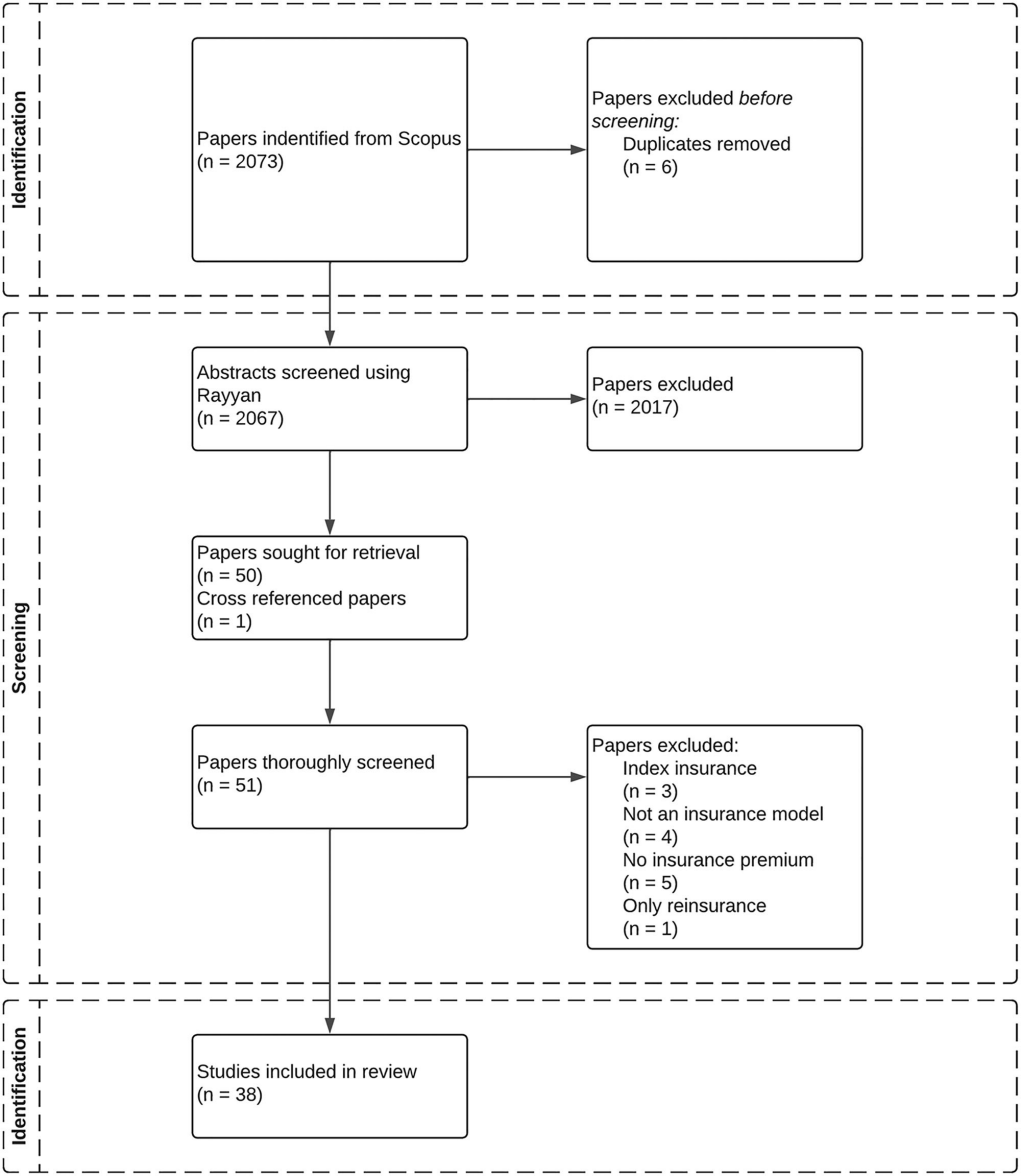
Selection process of papers included in the review.

For this paper, a systematic literature review process was conducted following the PRISMA guidelines and building on existing reviews.^22^, ^25^ First, keywords for querying papers were selected. Second, papers were queried within a literature database (Scopus). Third, the queried papers were screened for their suitability. The process is shown in a PRISMA flowchart (Figure 1).

#### Keywords

For our review, we have addressed three keyword types: “hazard‐related keywords,” “model keywords,” and “insurance keywords.” Using combinations of the three keyword types in the query with “AND” and “OR” Booleans ensures that only papers with abstracts that mentioned a hazard type, a model type, and an insurance‐related word were selected. This action was undertaken with the intent of refining the query to encompass papers within the area of interest. However, to make the query more exhaustive, the keywords were often kept a bit broader. For example, in the hazard type keyword list, words, such as “disaster,” were also chosen. The selected keywords for the hazard, model, and insurance types are summarized in Table S1 (online only).

#### Query

The “advanced search” function of Scopus was used to query the papers. We used Scopus because it was often used in similar literature reviews.^22^, ^26^, ^27^ First, the potential search strings were tried to obtain several papers that were large enough to contain all the suitable papers but small enough to be feasible. Keywords consisting of multiple words were put between quotation marks to make sure Scopus would only look for instances where the entire keyword was present. The language was limited to English, and the document type was limited to papers. The query was carried out for the title, abstract, and keywords of each paper. The final query had 2073 hits (of which 6 were duplicates), which is comparable to similar reviews.^25^ The search string used can be found in the Supporting Information Appendix (online only). One paper^28^ was added to the selection via cross referencing.

#### Screening

In the last step, the 2067 papers selected by Scopus were screened using Rayyan.^29^ Because the review focuses on the state of the art of insurance modeling, papers published before 2010 are excluded. Additionally, papers related to index or parametric insurance contracts were excluded because these types of insurance are deemed too dissimilar to the insurance under consideration in this study. After the manual screening, 50 papers were selected for a thorough review. During this process, three papers were deselected because they were about index insurance, four papers were excluded because they did not use an insurance model, five papers were deselected because they did not compute an explicit insurance premium, and one paper was excluded because it considered only a reinsurance model instead of an insurance model. This left 38 papers for the final analysis. The 13 deselected papers can be found in Table S2 (online only), with the accompanying reason for exclusion.

## RESULTS

### Model type

**FIGURE 2:**
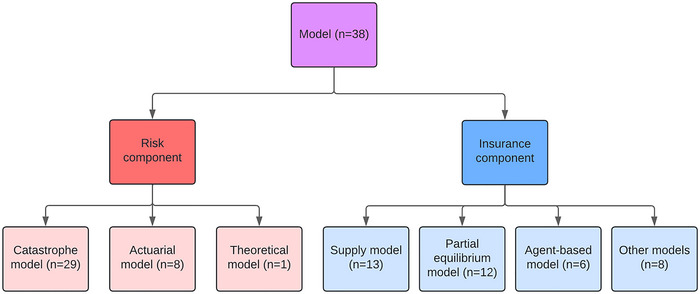
The number of papers per model type.

This section summarizes the findings about the risk model type and insurance model type of the reviewed papers. Figure 2 provides an overview of the number of papers per model type. For information about the model types per reviewed paper, refer to Table S3 (online only).

#### Risk model type

The commonality among all papers in this review is that they make use of a model to compute an insurance premium based on a climatic risk. Based on our review, we distinguish three methods of operationalizing risk: catastrophe modeling, actuarial modeling, and theoretical modeling. These three methods are discussed below.

##### Catastrophe models

In catastrophe modeling, risk is simulated by combining information on hazard impacts and associated occurrence probabilities with the exposed elements at risk and their vulnerability.^30^ Often, hazard impacts and probabilities enable the construction of exceedance probability curves, which illustrate the likelihood of a loss surpassing specific threshold values. However, there are also more simplified catastrophe models that only combine hazard footprints (e.g., flood extent, windstorm field, or areas subject to heatwaves) with exposure data on building infrastructure to estimate risk without addressing the probability of such events.^30^ Most papers employ a catastrophe model because risk related to high‐impact low‐probability hazards is impaired by a lack of available observed loss data due to this low probability of occurrence. Hence, hazards, such as flooding and earthquakes, are mostly estimated via catastrophe modeling,^18^, ^31^, ^32^, ^33^, ^34^, ^35^, ^36^ but see Sidi et al.^37^ for a counterexample. An alternative rationale for the frequent use of catastrophe models is their ability to flexibly accommodate future climatic and socioeconomic conditions. A potential drawback of catastrophe modeling is that it requires an often computationally expensive multi‐layered approach with data on hazard probabilities, exposure, and vulnerability (e.g., Boudreault et al.,^34^ de Ruig et al.,^38^ or Ermolieva et al.^39^). The resulting outputs of a catastrophe model (loss or risk) can be plotted in a spatial manner using maps showing risk per pixel or per administrative unit.

##### Actuarial models

A subset of papers uses an actuarial base for their insurance model. Instead of being simulation based as with catastrophe models, actuarial models estimate risk based on actual events with loss data.^40^ Actuarial models are mostly applied to windstorm^41^ and wildfire hazards.^42^, ^43^, ^44^, ^45^ Using empirical loss data, the risk can be estimated using econometric methods such as regression. Examples are models that used the expected annual average burned area per municipality based on historical fire occurrences and the annual average burned area^44^ and models that used regression papers to estimate wildfire risk based on socioeconomic, geographical, and climate‐related variables.^42^ El‐Adaway^41^ showed that actuarial models can be combined with bootstrapping to enhance loss observations; in this application, three datasets of 5000 observations were created from 2000 actual windstorm observations. An advantage of an actuarial approach is the possibility to elucidate potential trends that do not yet have a physical understanding.^40^ On the other hand, given the high‐impact low‐probability nature of climatic disasters such as flooding, there is often a lack of historical data on these events to apply a statistical analysis (but see Islam et al.^46^ for an actuarial model applied to flooding).

##### Theoretical models

One reviewed paper does not apply its model to a case study.^47^ This model treats risk as a simple stochastic variable. Therefore, the model does not simulate risk using an underlying catastrophe model or based on empirical data. We classify this model as a purely theoretical model, as there is no underlying risk model specified.

#### Insurance model type

When the climatic hazard is operationalized as a risk via either catastrophe modeling, an actuarial approach, or a probabilistic/theoretical approach, the estimated risk can be used in an insurance model. We distinguish four types of insurance modeling: insurance supply models, partial equilibrium models, agent‐based models (ABMs), and “other models,” which comprise model types that are less prevalent in the literature. These four model types are discussed in this section.

##### Insurance supply models

The most common insurance application is the insurance supply model. An insurance supply model concerns the pricing of insurance contracts. An example of such a model is applied in Aerts and Botzen,^18^ which calculated the future evolution of risk‐based premiums for flooding in the Netherlands using a catastrophe model, considering several socioeconomic and climate scenarios. The premium was calculated per administrative area based on its expected annual damage (EAD) divided by the number of houses per administrative area. This risk estimate, together with a loading factor that represents the operational costs of providing insurance as well as a profit margin, provided an estimate of the premium per household. Using this method, a stark increase in insurance premiums over time was found due to climate change and socioeconomic developments and the fact that the uncertainty around these future developments complicates the insurers’ rate‐setting of long‐term contracts. Another example is Brunette et al.,^43^ who estimated premiums for multi‐hazard forest insurance using an actuarial approach in combination with an insurance supply model. With this method, it was found that the most efficient procedure is to assume independence between the natural hazards.

Most insurance supply models incorporate spatially explicit, risk‐based premiums, relying on catastrophe models or actuarial methods to assess spatial risk.^18^, ^34^, ^41^, ^44^, ^45^, ^48^, ^49^, ^50^, ^51^ Generally, the premium's spatial resolution is limited by the complexity of the underlying risk module. Some models calculate premiums with a high spatial resolution,^34^ in which premiums are calculated per individual house, aiming to explore methods for mitigating adverse selection. Calculating risk‐based premiums at a high resolution has the advantage of accurately reflecting the risk of the area and potentially incentivizing adaptation effort. However, risk‐based premiums can lead to unaffordability in high‐risk areas and may influence location decisions.^5^ This means that a pricing application alone is often not enough to answer all insurance‐related challenges. More intricate insurance applications, such as partial equilibrium models, not only compute premiums but also leverage these premium data in subsequent modules to, for example, obtain insights into insurance demand or adaptation efforts.

##### Partial equilibrium models

Partial equilibrium models assess equilibrium conditions in a particular market *ceteris paribus*.^52^ There are no feedback effects that alter the fundamental supply and demand relationships defined in advance.^53^ A partial equilibrium application is useful for determining equilibrium outcomes in an insurance market or considering the effect of insurance on equilibrium conditions between marginal cost and marginal revenue for a business.

By simulating insurance market conditions, insights about insurance uptake, such as the uninsured portion of risk or the unaffordability of insurance, can be obtained. These insights are showcased by studies on the European flood insurance market. An example is Tesselaar et al.,^19^ who found that insurance unaffordability will increase due to climate change and socioeconomic development by simulating premium prices and insurance demand for various scenarios. Another area in which partial equilibrium applications prove useful is when the effect of insurance on (agri) businesses is considered. For example, Brunette et al.^47^ analyzed the effect forest insurance can have on the implementation of adaptation efforts by examining the marginal cost and benefit of insurance in different situations. Results showed that including adaptation efforts in forest insurance contracts is a beneficial tool to promote adaptation efforts, especially if the type of adaptation effort is unobservable to the insurer. In a similar study, Barreal et al.^42^ examined the effect of insurance on the net present value of forest investments by analyzing the equilibrium between marginal risk reduction cost and benefit. Results showed that insurance plays a larger role in increasing the net present value of forest investments when restoration costs are included in the insurance policy. When insurance supply systems are considered, a partial equilibrium application can also be used to compare different insurance supply systems on key characteristics such as premiums and demand.^19^, ^28^, ^31^ Thereby, the model can be used to obtain insights into the desirability of insurance market reforms through evaluating both their supply and demand side effects.

##### Agent‐based insurance models

ABMs subdivide complex systems into a flexible simulation framework of individual autonomous, heterogenous, and active components (agents), which is useful for investigating complex and emerging agent behavior.^54^ ABMs offer valuable insights for climate risk insurance modeling by simulating the intricate interactions and dynamic behaviors among consumers and insurers in the market, thus providing valuable insights into (emerging) consumer behavior. It is noteworthy that all papers with an ABM application consider flood insurance. One example is a study by Dubbelboer et al.,^21^ which applied an ABM to simulate the UK housing market to assess the viability of the FloodRe scheme. Another example is a study on the US flood insurance system by de Ruig et al.,^33^ which investigated the societal benefits of risk‐based premiums in a changing climate. The type of consumer behavior that is modeled in ABMs usually comprises insurance uptake,^21^, ^33^, ^38^, ^55^, ^56^, ^57^ implementing disaster risk reduction (DRR) methods,^21^, ^33^, ^38^, ^55^, ^56^ and the decision to purchase property.^21^, ^55^, ^56^, ^57^

The interactions in the ABMs depend on the modeled agents and the focus of the model. Interactions are commonly modeled between consumers and the development of risk^33^, ^38^ or impact^21^, ^55^, ^56^ of a flood event. Households base their decision to take DRR measures or insurance on the severity of the risk they face.^33^, ^38^ In other cases, the decision to take DRR measures is based on whether a flood event occurred.^21^, ^55^, ^56^

Another common interaction addressed in ABMs is an interaction between households and the insurance market. In studies by de Ruig et al.,^33^, ^38^ households decide each year whether to purchase insurance or not. This decision is linked to a subjective expected utility function that takes the (risk‐based) premium calculated by the insurance sector, the budget of the household, and a deductible into account. In some ABMs, households are mandated to take flood insurance but can influence their premium by moving to another location or by undertaking DRR measures.^21^, ^55^, ^56^ In Tanaka et al.,^57^ households decide whether to move or not based on a utility function that considers flood risk reflected by the insurance premium.

Allowing for individual agent behavior is useful concerning the implementation of DRR measures.^21^, ^33^, ^38^, ^55^, ^56^ Furthermore, modeling interactions between consumers and the insurance market leads to useful insights about insurance uptake and affordability.^33^, ^38^ Another strength of an ABM is its suitability for integrating climate change and socioeconomic development scenarios. This is also reflected in the fact that all reviewed ABMs include at least one climate change scenario. Moreover, because an ABM often includes data on the characteristics of agents such as income, socioeconomic development scenarios are often applicable.^33^, ^38^, ^57^

##### Other insurance model types

There are two other insurance model types that can be distinguished in the literature, insurance demand models^46^, ^58^ and game‐theoretic models.^35^, ^59^, ^60^, ^61^ The goal of an insurance demand model is to obtain an insight into the demand for insurance. Birghila et al.^58^ did this by analyzing the optimal risk layering of insurance contracts per recipient to maximize uptake under ambiguity. Islam et al.^46^ analyzed the willingness to pay for insurance via a logit model based on a field survey. Another insurance model type is a game‐theoretic model. A game‐theoretic model shares similarities with an ABM but places a greater emphasis on equilibrium conditions and optimization.^62^ Utilizing game‐theoretic models proves useful in capturing the dynamics between the demand and supply sides of insurance. This framework offers valuable insights into the strategic choices made by both insurers and insurance consumers. An example is Peng et al.,^35^ who highlighted the existence of policies that include retrofitting and make all actors (households, government, insurers, and reinsurers) better off than a policy that does not include retrofitting. Game‐theoretic models can serve as a valuable tool for analyzing the wider implications of insurance, retrofitting initiatives, and the acquisition of high‐risk properties on overall losses.^60^

### Risk

Risk can be subdivided into hazard, vulnerability, and exposure,^63^ where the hazard is defined as the frequency and intensity of the natural hazard, exposure as the presence of exposed values, such as buildings, property, or crops that can adversely affected, and vulnerability as the susceptibility of these exposed values to losses.^5^ This section reviews the modeling input referring to the risk component of the model. Details about the risk component per reviewed paper can be found in Table S4 (online only).

#### Hazard

In this paper, we identify five climatic hazard groups: flooding, wildfires, hurricanes, windstorms, and other hazards.

Flooding is overrepresented in the literature, with more than half of the papers being applied to flood hazards. We further divide flood hazards into three subcategories: riverine flooding,^18^, ^19^, ^20^, ^28^, ^31^, ^32^, ^33^, ^34^, ^37^, ^39^, ^48^, ^57^, ^59^, ^64^ coastal flooding,^18^, ^33^, ^38^, ^39^, ^51^ and other flooding (which consists of pluvial flooding,^57^ surface water flooding,^21^, ^55^, ^56^ flash floods,^46^ and flooding in general).^36^ Of these types, riverine flooding accounts for more than half of the flood modeling papers. In some cases, a combination is used between riverine flooding and another type of flooding.^18^, ^33^, ^39^, ^57^ Moreover, coastal flooding is used in all but two cases,^38^, ^51^ in combination with riverine flooding. Of the other hazard types, hurricanes/cyclones^35^, ^60^, ^61^, ^65^, ^66^ and wildfires^42^, ^43^, ^44^, ^45^, ^50^, ^67^ occur the most. To a lesser extent, there are models about windstorm insurance.^41^, ^50^, ^68^ The group “other hazards” consists of forest‐related damages,^43^, ^47^ earthquakes,^36^ debris flows,^69^ drought,^58^ and natural disasters in general.^49^

Most of the reviewed papers tend to employ models that exclusively focus on addressing individual natural hazards. Models with a multi‐hazard approach do exist, for forestry‐related damages,^43^, ^50^ and for flooding and earthquake damages.^36^ Perazzini et al.^36^ explicitly used both a single‐hazard and a multi‐hazard insurance policy in their case study. The low attention to multi‐hazard insurance indicates a gap in the climate insurance modeling literature: Compound climate risks are increasing rapidly, and an expanding literature focuses on multi‐hazard climate risk assessments,^1^ but multi‐hazard risks are not often considered in climate insurance models.

The way in which the hazard is operationalized varies by hazard group and risk model type. For flooding, the hazard is commonly determined as the inundation extent with a certain return period in a certain grid cell or area.^18^, ^19^, ^20^, ^21^, ^28^, ^31^, ^32^, ^33^, ^38^, ^48^, ^55^, ^56^, ^57^ This means that inundation depths are linked to a certain probability each year per grid cell or area. This probability and inundation depth can then be used in combination with exposure and vulnerability data to estimate the EAD. Concerning hurricanes, three of the reviewed models^35^, ^60^, ^61^ all used a set of probabilistic hurricane scenarios based on historical records.^70^ These hurricane scenarios comprise a track with certain parameters that determine the intensity and probability of occurrence. Kunreuther et al.^66^ similarly used hurricane scenarios but also included future scenarios. For wildfires, the hazard is usually determined with an econometric model and depicted as a probability per area based on historical wildfire occurrence.^42^, ^44^, ^50^ In Kunreuther et al., which uses a catastrophe model, the probability of a household being affected by a wildfire is based on a simulation and varies based on the number of houses in vicinity. These probabilities are more often denoted by region rather than grid cell, in contrast with flooding. Similar to the assessment of wildfires, the evaluation of windstorm hazard usually relies on historical data analysis^41^, ^50^ (but see Loisel et al.,^68^ which uses return periods).

#### Exposure

For flooding, exposure is commonly operationalized via data about land use.^18^, ^20^, ^21^, ^28^, ^31^, ^32^, ^33^, ^34^, ^38^, ^39^, ^51^, ^55^, ^56^, ^57^, ^64^ However, there are differences in the resolution of this approach. For example, although it is common to use aggregated information about land use, data for single houses, including characteristics, such as number of floors and main usage, can also be used.^34^ Furthermore, forward‐looking models often include GDP growth and population growth as a proxy for the growth in exposure.^18^, ^20^, ^28^, ^31^, ^32^, ^33^, ^38^, ^57^, ^64^

In hurricane‐focused insurance models, there is a heavier focus on residential buildings than in insurance models for flooding. Hence, the approach is less land‐use‐based and more focused on the buildings themselves. Exposure can be aggregated by building class^35^, ^60^, ^61^ or be based on the value of assets in an insurance portfolio,^66^ or the value per building.^65^

Papers considering wildfire insurance models are mostly forestry related (but see Kunreuther et al.^67^ and Thompson et al.^45^ for non‐forest insurance). This means that exposure input data for these models are related to forest stand value.^42^, ^43^, ^47^, ^50^ It is common to relate this value to the age of the forest stand.

Concerning windstorm insurance models, the approach is similar to wildfire insurance models, as both categories are mostly applied to the forestry sector.^43^, ^68^

#### Vulnerability

For flooding, vulnerability is commonly depicted by depth–damage curves.^18^, ^19^, ^20^, ^21^, ^28^, ^31^, ^32^, ^34^, ^39^, ^48^, ^55^, ^56^, ^64^ A depth–damage curve relates inundation depth to monetary damage.^71^ In this manner, vulnerability can be operationalized by assigning distinct depth–damage curves to various buildings or land‐use categories. For riverine and coastal flooding, protection standards, such as dykes and levees, are often considered.^18^, ^20^, ^28^, ^31^, ^32^, ^64^

Concerning hurricanes, Kunreuther et al.^66^ differentiated between two vulnerability conditions, one with adaptation standards compliant with local building codes and one with the current observed adaptation standards. Similarly, Walker et al.^65^ differentiated between two vulnerability conditions: current practice and more stringent design. Other examples include modeling the building resistance level as a parameter and dividing buildings into classes based on location and category.^35^, ^60^, ^61^

Because wildfire insurance models are mainly targeted to forestry insurance, modeling input concerning vulnerability to wildfires is also mostly targeted to forestry practices. One way in which vulnerability is translated for the forestry sector is as a forest management parameter. This parameter stands for the level of preventative measures that are taken and is inversely related to the risk.^42^ Another paper makes use of empirical vulnerability functions based on the age class of the trees and the probability of destruction.^43^

Similar to wildfires, windstorm vulnerability is also mainly targeted to forestry insurance. The empirical vulnerability functions in Brunette et al.^43^ are also applied to windstorms. Concerning trees, the effect of age on vulnerability is more apparent for windstorms than for wildfires.^68^ Loisel et al.^68^ operationalized this vulnerability by examining age‐dependent tree characteristics, specifically diameter and height. They posited that an increase in the percentage of damaged trees occurs when these characteristics attain higher values.

#### Location

There is only one paper that did not apply its model to a location‐based case study.^47^ Nearly half of the reviewed papers applied their model to a case study that takes place in Europe.^18^, ^20^, ^21^, ^28^, ^31^, ^32^, ^36^, ^39^, ^42^, ^44^, ^47^, ^50^, ^55^, ^56^, ^58^, ^64^, ^68^ Of the remaining papers, most of the case studies take place in the United States.^33^, ^35^, ^38^, ^45^, ^51^, ^60^, ^61^, ^66^, ^67^ A few papers feature a case study in Asia.^37^, ^46^, ^49^, ^59^, ^69^ Two case studies take place in Canada.^34^, ^48^ One case study is applied to Australia.^65^ The predominant pattern here is that climate risk insurance models are most often applied to western and developed countries compared to less‐developed countries. For instance, no model is applied to Africa or South America, which are areas that are more vulnerable to climatic hazards^1^ and might, therefore, benefit from insurance coverage. A possible explanation for the lack of case studies in these regions is the requirement of high‐level input data, which are often harder to acquire in less‐developed countries. Innovations in the usage of satellite imagery might offer a solution for this problem.^46^

#### Scenarios

Models that are forward‐looking use projections of how the risk develops over time. This is often done by using a climate change scenario in the risk component of the model. Due to the uncertainty of climate change, it is common to use multiple climate change scenarios in estimating future natural disaster risk.

About half of the reviewed papers can be classified as forward‐looking. These papers considered at least one climate change scenario in their approach. The scenarios considered are often the Representative Concentration Pathways (RCPs). RCPs are radiative forcing trajectories until 2100 for different climate change scenarios, ranging from 2.6 to 8.5 W/m^2^.^72^ These trajectories can be employed to simulate future climate conditions in a model and, if multiple RCPs are used, compare the model under different climate change scenarios. If multiple climate change scenarios are employed, such as a low RCP and a high RCP, it becomes possible to set a lower and upper bound on the possible outcomes of a model, capturing the uncertainty around climate change. There are, however, several papers that can be considered forward‐looking but only employ one climate change scenario.

More than half of the papers that applied a climate change scenario to their model also applied a socioeconomic development scenario. There are no instances where only a socioeconomic development scenario is applied. The socioeconomic development scenarios often used are the Shared Socioeconomic Pathways (SSPs). SSPs describe different socioeconomic development trajectories such as sustainable development and fossil‐fueled development.^73^ The SSP2 (middle of the road) and SSP5 (fossil‐fueled development) scenarios are often paired with the RCP4.5 and RCP8.5 scenarios, respectively, as they have similar traits.^19^, ^31^, ^32^, ^33^ Another way in which socioeconomic development scenarios are being used is in the form of simulating future land use.^18^, ^19^, ^31^, ^32^ Tanaka et al.^57^ incorporated income and house prices that increase over time, reflecting a constant economic growth rate.

#### Adaptation

Adaptation (often referred to as DRR) measures to reduce climate risk are often accounted for. More than half of the reviewed papers include some form of DRR. Often, these papers employ a forward‐looking model by means of a climate change scenario, as modeling adaptation measures is especially interesting for forward‐looking models.

In reality, adaptation is usually financed by governments and consumers of insurance. Of the papers that included adaptation, most did so for adaptation financed by households^20^, ^31^, ^32^, ^38^, ^65^, ^66^ or by both households and the government.^21^, ^28^, ^33^, ^35^, ^56^, ^60^ A subset of the reviewed papers included adaptation measures financed by agribusinesses.^42^, ^47^, ^58^ Furthermore, Aerts and Botzen^18^ and Unterberger et al.^64^ considered adaptation financed by only the government. Models that consider insurance against wildfires and/or storms generally did not include adaptation (but see Barreal et al.^42^ and Kunreuther et al.^67^). However, adaptation measures against these hazards do exist.^74^, ^75^

Adaptation measures have the potential to reduce natural disaster risk. When a premium is risk‐based, investing in adaptation measures can potentially lower this premium. This not only incentivizes proactive risk management but also promotes broader societal engagement in resilience‐building efforts. This ultimately fosters a more economically viable and secure environment for both insurers and policyholders. An important question is how insurance arrangements can incentivize investment in risk reduction measures.^5^ The idea of using insurance to stimulate adaptation is explored in multiple papers and across hazard type.^20^, ^35^, ^47^, ^56^ For example, Hudson et al.^20^ showed that correctly incentivizing adaptation via insurance can lead to a reduction in household flood risk of 12% in Germany and 24% in France by 2040.

### Insurance

This section summarizes findings about the insurance component of the model. Details per reviewed paper can be found in Table S5 (online only).

#### Recipient and the decision to insure

About two‐thirds of the reviewed studies concern insurance for households. Two papers included insurance for households in combination with insurance for another entity; a model where both households and the government are insured^59^; and a model where both households and firms are insured.^39^ Furthermore, some papers modeled insurance for structures such as civil infrastructure developments^41^, ^64^ or insurance for buildings in general.^37^ Multiple papers modeled agricultural insurance, of which two papers concerned some form of crop insurance,^46^, ^58^ and six papers focused on insurance for forestry.^42^, ^43^, ^44^, ^47^, ^50^, ^68^ Although agricultural insurance can be considered insurance for firms, Ermolieva et al.^39^ is the only paper that considered insurance for general firms alongside households by using land‐use maps.

Models that are not only supply‐focused also often incorporate a consumer decision component. This decision component indicates, if applicable, the way in which the decision to purchase insurance is made. Insurance uptake can be summarized into two categories: mandatory uptake and voluntary uptake. Concerning mandatory uptake, the premium can be risk‐based when a solidarity market structure is concerned.^19^, ^28^, ^31^, ^32^ There are also examples of papers that use mandatory uptake but do connect the premium to the risk. These papers either assume that all constituents purchase insurance^18^, ^21^, ^55^, ^56^ or a given percentage of households.^57^ For voluntary uptake, the decision to insure is based on expected utility maximization. In this way, the insurance recipient (commonly households) makes the decision based on a (subjective) utility curve. In essence, the insurance recipient determines whether acquiring insurance provides greater value than not obtaining insurance by weighing the prospective loss against the premium payment. The way in which this decision method is employed varies mostly in the degree of rationality that is assumed. In Kesete et al.,^61^ the insurance recipient is assigned a risk aversion coefficient based on the risk region but has no specific rationality constraint. This differs from the model employed by, for example, Hudson et al.^28^ and Tesselaar et al.^19^ where a subjective expected utility framework is used. The subjective expected utility framework incorporates variations in risk perception from the objective risk to account for bounded rationality. Subjective expected utility is also used in studies where households are assumed to overestimate their risk after a flood and underestimate their risk after a period of no floods.^33^, ^38^ Other models make use of an expected utility curve but do not include a rationality constraint.^35^, ^47^, ^69^

#### Insurance sector modeling

Climate risk insurance is organized differently across countries and hazard types.^76^ Moreover, insurance can be arranged privately, publicly, or a combination of the two.^28^

Insurance supply models predominantly concentrate on the pricing aspect of an insurance contract and typically omit explicit consideration of the insurer as an agent (e.g., Boudreault et al.^34^; Brunette et al.^43^; Sacchelli et al.^50^). More often, the insurer as an agent is incorporated, but only one representative insurer is assumed to exist (e.g., Kalfin et al.^49^; Birghila et al.^58^; Kesete et al.^61^). Not including an insurer as an agent or assuming the insurer to be a single agent is a common modeling assumption. This assumption is also frequently employed in models focused on insurance demand.^46^, ^58^ An alternative format involves modeling an insurance market wherein a public entity assumes the role of providing insurance, as opposed to a private company. This approach is frequently employed in partial equilibrium and ABMs, where multiple market forms are simulated and considered.^21^, ^28^, ^38^, ^55^, ^56^

Another representation of the insurance sector is delineating the insurance component as a public–private market, wherein the government assumes the role of a risk‐neutral reinsurance agent providing support to insurers (e.g., Perazzini et al.^36^; Hudson^28^; Aerts and Botzen^18^) or a publicly organized insurance market in which a public agent provides insurance instead of a private company (e.g., Crick et al. ^55^; de Ruig et al.^38^).

Certain models offer an evaluation of the effectiveness of diverse insurance structures, spanning from private to public configurations. Hudson et al. ^28^ evaluated six different insurance systems in the EU on their ability to cope with trends in flood risk and found that introducing elements of public–private partnerships can improve the affordability of insurance. In a study conducted by Unterberger et al.,^64^ three distinct insurance systems are analyzed with regard to their fiscal impact on forthcoming governmental budgets and the associated variability in disbursements for public infrastructure insurance. As another example, Kunreuther et al.^66^ differentiated between hard and soft insurance market conditions to evaluate how the supply system for hurricane insurance behaves under these different conditions. In a similar fashion, Tesselaar et al.^31^ analyzed the effect of climate change on premiums, affordability, and insurance uptake in soft and hard reinsurance conditions.

A select number of models integrate the consideration of insurer competition, each employing distinctive methodologies in their approach. One model type that is well suited for modeling competition is the game‐theoretic model type. For example, Guo et al.^60^ simulated multiple insurers that participate in a perfect information Cournot–Nash noncooperative game to calculate the premium. Another way in which competition is considered is by assuming Bertrand competition among the insurers. This is done by omitting a premium profit margin (e.g., Hudson et al.^28^; Tesselaar et al.^31^; Kalfin et al.^49^), indicating that the insurers cannot earn a high profit due to the market being competitive. A general observation is that none of the reviewed ABMs explicitly model competition among insurers. Most ABMs either use a public insurance agent^33^, ^38^ or assume only one insurer in the model.^21^, ^55^, ^56^ Tanaka et al.^57^ employed an ABM that does not include an insurer as agent but does calculate an insurance premium.

#### Premium calculation

Insurance premiums are often computed using various methodologies, with a prevalent approach being the adoption of a risk‐based premium. This type of premium is designed to mirror the inherent risk associated with the insured entity. The utilization of risk‐based premiums holds significance, as it facilitates alignment between premium revenue and projected indemnity disbursements, thereby contributing to the financial viability of an insurance scheme. Furthermore, the deployment of risk‐based premiums serves as a means to convey information pertaining to risk.^77^, ^78^ Additionally, these premiums can serve as a mechanism to incentivize the implementation of DRR measures.^77^

An example of a model that uses risk‐based premiums for wildfires can be found in the study by Sacchelli.^50^ For risk‐based insurance against hurricanes, models by Kunreuther et al.^66^ and Walker et al.^65^ can serve as examples. Alternative methods for computing insurance premiums include the application of the distortion premium principle.^58^ Another approach involves representing the premium as a random variable.^37^ Additionally, a quantile‐based methodology offers an alternative perspective on premium calculation.^39^ In some models, premiums are determined via aggregated risk in a solidarity market.^19^, ^28^, ^32^

Studies addressing multiple insurance supply systems frequently employ diverse methodologies in premium calculation. An illustration of this multifaceted approach is evident in the work of Hudson et al.,^28^ where premiums are determined through various models. These models encompass scenarios where premiums are unrelated to risk, fully risk‐based, or risk‐based with an imposed cap. A similar instance is illustrated in the research conducted by de Ruig et al.,^33^ where the determination of premiums varies across several approaches. These include premium calculations based on outdated risk maps, fully risk‐based assessments, computations grounded in updated risk maps following a flood event, and premiums derived from periodically updated risk maps. The adoption of diverse premium calculation methods proves to be a valuable strategy, facilitating comparisons among distinct market types or risk assessment methodologies. This comprehensive approach contributes to novel insights within the field of climate risk insurance modeling. As an example, by performing a multi‐criteria analysis on different insurance market types, Hudson et al.^28^ found that a public–private partnership system can reduce the unaffordability of insurance by performing a multi‐criteria analysis on different insurance market types.

## DISCUSSION

### State of the art and directions for future research

Climate risk insurance models can be subdivided into two components: the risk module and the insurance module. The shape of these components generally depends on the model type, the climatic hazard, and the application of the model. In terms of model type, we distinguish three primary categories: insurance supply models, partial equilibrium models, and ABMs.

Insurance supply models are useful for premium calculations, which may include premium development over time under different socioeconomic development and climate change scenarios (e.g., Aerts and Botzen^18^; Boudreault et al.^34^). However, most supply models are not forward‐looking (e.g., Boudreault and Ojeda^48^; Brunette et al.^43^; El‐Adaway^41^; Sacchelli et al.^50^). Partial equilibrium models allow for analyzing the interplay between insurance supply and demand. This makes partial equilibrium models useful for insurance market type assessments (e.g., Hudson et al.^28^) or for investigating inquiries pertaining to the affordability and uptake of insurance (e.g., Tesselaar et al.^19^). ABMs allow for the simulation of complex agent behavior. This is useful to analyze adaptation decisions that reduce climate risk (e.g., de Ruig et al.^33^; Dubbelboer et al.^21^; Jenkins et al.^56^).

The risk component of a model estimates the risk used to calculate an insurance premium. This component can (with one exception)^47^ be divided into catastrophe models and actuarial models. Most of the reviewed papers estimate risk using a catastrophe model. A catastrophe model simulates the risk based on hypothetical events and is, hence, useful for estimating the risk of low‐probability high‐impact events such as flooding. On the other hand, actuarial models use loss data about actual events to estimate the risk. Therefore, the actuarial approach tends to be more applicable to hazards that happen more commonly such as windstorms.

The insurance component of the model translates the risk into an insurance application. Although most papers focus on household insurance, it is worth noting that forestry insurance modeling is also a well‐established and developed field.^42^, ^43^, ^44^, ^47^, ^50^, ^68^ Multiple papers include a modeled insurer, often in ABMs,^21^, ^33^, ^38^, ^55^, ^56^ partial equilibrium models,^19^, ^20^, ^28^, ^31^, ^32^, ^36^, ^39^, ^47^, ^64^, ^67^ or game‐theoretic models.^35^, ^60^, ^61^ The premium calculation predominantly follows a risk‐based approach, wherein the premium is designed to mirror the level of risk inherent to the insured entity. The usage of risk‐based premiums is common for climate risk insurance.

More than half of the papers about climate risk insurance models capture flood hazards. This means that the other climatic hazards are relatively underrepresented in the literature. Insurance for climatic hazards such as drought and windstorm damage tends to be relatively understudied in comparison to flooding. This is despite windstorms accounting for a substantial 40% of the total losses attributed to climate‐related events, whereas flooding constitutes 25%.^2^ The disproportionate attention to flood‐related research compared to the distribution of overall losses highlights an imbalance in the focus on various climatic perils.

Another key research gap is the application of climate risk insurance models to underdeveloped countries. Of the models considered, only a small subset is applied to Asia, and none of the models are applied to locations in Africa or South America. A potential reason for this is the lack of available data. However, because these areas are relatively more vulnerable to climatic hazards than most developed areas,^1^ insurance and, therefore, insurance modeling are relevant there. Utilizing remote‐sensing techniques to assess the risk for insurance purposes^46^ can potentially prove useful in locations where data collection is difficult.

A small subset of models concerns insurance for the commercial sector, and of this small subset, all papers except for Ermolieva et al.^39^ considered only agribusinesses. However, the commercial sector also experiences substantial damage from climatic hazards due to direct impact and business interruptions as a consequence of these direct impacts. Business interruptions can have significant and widespread consequences, potentially resulting in outcomes such as unemployment and product shortages.^79^, ^80^, ^81^ Therefore, amidst a shifting climate, it is imperative to assess the feasibility and resilience of climate risk insurance for businesses. Achieving this goal will necessitate increased modeling efforts within this domain.

A significant research gap exists in the observation that merely half of the climate risk insurance models can be categorized as forward‐looking. This implies that only half of these models integrate future scenarios to evaluate insurance mechanisms in the context of a changing climate and evolving socioeconomic development scenarios. Evaluating risk based on the experience from past events is no longer sufficient to capture the uncertainties around future risks.^10^ Future premium setting is impeded by the uncertainty around climate change.^5^ This calls for a thorough forward‐looking approach to climate risk insurance setting, which is currently not happening enough, indicating a research gap. This gap is evident for both the inclusion of climate change scenarios, such as RCP scenarios, and the inclusion of socioeconomic development scenarios, such as SSP scenarios. Furthermore, not all papers that are categorized as forward‐looking include multiple scenarios. Utilizing multiple scenarios enables a more comprehensive capture of the uncertainties associated with the future.

Furthermore, wildfire and windstorm insurance models often do not include adaptation (but see Barreal et al.^42^ and Kunreuther et al.^67^). Even though adaptation for these perils does exist,^74^, ^75^ the incorporation of adaptation into insurance models for these climatic hazards remains limited. Consequently, there is a potential avenue for enhancing the robustness of insurance models by integrating adaptation elements specific to wildfire and windstorm risks.

Another gap is the limited attention to multi‐hazard modeling. Multi‐hazard risk modeling is an emerging field that poses a more thorough approach to risk management than traditional methods.^82^, ^83^ Currently, there is a small number of papers that specifically consider multi‐risk premiums, and they either consider forestry insurance policies^43^, ^50^ or household insurance against earthquakes and flooding.^36^

### Policy recommendations

Most forward‐looking models indicate that climate change and socioeconomic developments highly exacerbate future risk and, hence, lead to increased insurance premiums.^19^, ^20^, ^21^, ^31^, ^32^, ^34^, ^55^, ^56^, ^64^, ^66^ This suggests that taking climate change and socioeconomic developments into account in insurance models is imperative in assessing the long‐term viability of insurance. However, uncertainty about future risks gives some insurers an incentive to charge higher surcharges on insurance premiums and restrict coverage for extreme weather events.^5^ Applying a stochastic approach rather than a deterministic approach in climate risk assessment^65^ and taking the ambiguity among different climate models into account^58^ are methods to deal with this uncertainty.

Multiple papers advocate for the implementation of risk‐based premiums in natural disaster insurance schemes because they are useful for incentivizing adaptation efforts.^20^, ^21^, ^28^, ^33^, ^38^, ^47^, ^56^, ^64^, ^67^ For instance, risk‐based premiums may act as a price signal that raises awareness among policyholders of the climate risks they face. Moreover, rewarding policyholders who make their properties resistant to the impacts of extreme weather with premium discounts gives them a financial incentive for taking adaptation measures against climate risks. Incentivizing adaptation is also a recommendation given in a joint discussion paper by the ECB and the European Insurance and Occupational Pensions Agency (EIOPA).^24^ However, it is also important to consider the affordability of insurance, as fully risk‐based premiums might lead to unaffordability and, hence, a reduced uptake among low‐income households in areas with a high natural disaster risk.^19^, ^64^ A potential policy solution for this unaffordability might be the usage of a voucher scheme, which alleviates the share of the insurance premium that is considered unaffordable.

Furthermore, to address increasing climate risk and keep insurance schemes viable, a proactive involvement of the government in the insurance market has been proposed through the establishment of public–private partnerships.^19^, ^28^, ^31^ In such an approach, the government strives to reach a balance between ensuring the financial viability of insurance companies and keeping premiums affordable for the general public. Another example of this can be found in the research that actively examined improvements in the UK public–private partnership FloodRe.^21^, ^55^, ^56^ The government can also be involved by means of enforcing insurance uptake, thereby increasing the pool of policyholders. Pinheiro and Ribeiro^44^ and Tesselaar et al.^31^ suggested that the mandatory uptake of insurance can lead to higher resilience, the former for forestry businesses concerning wildfire hazard and the latter for households concerning flood hazard. Mandatory uptake leads to the possibility of spreading the risk across more policyholders, leading to lower premiums and a lower protection gap.

Lastly, multiple studies suggest that developing insurance products that cover multiple climate risks can be attractive for enhancing insurance coverage for climate risks.^28^, ^36^, ^43^, ^50^ This would require a move from single‐ to multi‐hazard climate risk assessments in insurance modeling. Combining multiple hazards under a single insurance policy has been observed to necessitate a lower amount of capital compared to insuring each hazard individually due to risk diversification.^36^

All these recommendations require close collaboration among stakeholders at different levels (e.g., ECB and EIOPA).^24^ Birghila et al.,^58^ Crick et al.,^55^ Hudson et al.,^20^ and Sidi et al.^37^ emphasized the importance of involving diverse stakeholders (e.g., government, other private partners) to create a more nuanced, effective, and transparent risk management framework. Collaboration between all stakeholders involved can limit uncertainty. Specifically, collaboration between the insurance and public sectors is often crucial.^84^ An example is a clear communication of the government about post‐disaster compensation to limit the crowding out of demand for private insurance, also called charity hazard.^32^ Furthermore, due to the inherent complexity of insurance products, collaboration between insurers and government stakeholders not only widens the spectrum of perspectives but also enhances the adaptability of insurance strategies to different challenges. Examples are combining private insurance coverage with public adaptation measures that limit climate risk and introducing public–private insurance coverage when premiums otherwise rise to unaffordable levels.

## CONCLUSION

This paper has synthesized the literature on climate risk insurance models and their characteristics. Climate risk insurance models range from simple pricing applications to more complex partial equilibrium and ABMs that can be used to assess research questions about insurance uptake and affordability. All models can be subdivided into two components: the risk and the insurance modules. The risk module can either be a catastrophe model that simulates the risk approached from hazard, exposure, and vulnerability aspects, or it can be based on historical data via an actuarial approach. Catastrophe models are typically more effective in assessing the risk of climatic hazards characterized by a low probability of occurrence but high impact, such as floods. On the other hand, actuarial approaches prove more beneficial in evaluating risks associated with climatic hazards that occur more frequently, such as windstorms or wildfires.

Most forward‐looking models indicate that climate change and socioeconomic developments exacerbate future risk and, hence, lead to increased insurance premiums. Various studies recommend introducing risk‐based premiums to incentivize adaptation efforts that limit this increase in climate risks, combined with policy strategies that address affordability issues among low‐income households. Other findings point toward introducing public–private insurance to cope with climate change and enhance risk spreading by introducing insurance purchase requirements or insurance products that cover multiple climate risks.

We identify knowledge gaps and suggest a research agenda that aims to improve modeling techniques to aid decision‐making in insurance policy design. First, flood insurance tends to be highly overrepresented in the climate risk insurance modeling literature. Second, most models are applied to case studies in developed countries, despite the potential for developing countries to experience a more substantial increase in natural disaster damages, making them potentially more significant beneficiaries of insurance coverage. Third, the coverage for non‐agricultural commercial sector insurance is limited, even though a sizable portion of the climate‐related damages can be found in this sector, also through business interruption.

Merely half of the reviewed papers applied forward‐looking climate risk analyses by utilizing climate change scenarios to examine the impact of climate change on risk. With climate change increasing the frequency and severity of natural hazards, this indicates a considerable research gap. Furthermore, an even smaller number of studies incorporated socioeconomic development scenarios to consider their effects on future risk. This suggests that only a subset of the reviewed papers is truly valuable for evaluating the ability of insurance to cope with future climate change.

The field of climate risk insurance modeling is growing, and the current state‐of‐the art models are certainly capable of addressing pivotal inquiries related to climate risk insurance. Addressing the research gaps identified by our review is imperative for delivering insights into how the insurance sector can proactively adapt to the challenges posed by climate change. By refining models, expanding geographical and hazard coverage, improving the inclusion of the commercial sector, and embracing a forward‐looking perspective, the insurance industry will be better equipped to fulfill its role in mitigating the financial impacts of climate‐related losses and fostering resilience.

## AUTHOR CONTRIBUTIONS

M.W.I. conducted the review and drafted the original manuscript. W.J.W.B., J.C.J.H.A., J.B., and M.T. contributed to writing, provided critical revisions, and supervised the work. All authors reviewed and approved the final manuscript.

## CONFLICT OF INTEREST STATEMENT

The authors declare no conflicts of interest.

## Supporting information



Supporting Information
