# Supplementary material for: The state of the art and future of climate risk insurance modeling
Source: Ann N Y Acad Sci. 2024 Nov 12;1541(1):100–14. doi: 10.1111/nyas.15255 (PMC11580769; doi:10.1111/nyas.15255)
Supplement: Supplementary file 1 — Supporting Information [file NYAS-1541-100-s001.docx]

**The State of the Art and Future of Climate Risk Insurance Modeling – Supplementary Material**

Michiel W. Ingels,^1^ Jeroen C.J.H. Aerts,^1^ W.J. Wouter Botzen,^1^ Jan Brusselaers,^1^ Max Tesselaar^1^

1. Institute for Environmental Studies (IVM), Vrije Universiteit, Amsterdam.

**Supplementary Table 1: Keywords used in queries and final SCOPUS search string**

| **Keyword group** | **Subgroup** | **Keywords** |
| --- | --- | --- |
| Hazard | Perils | flood, river, coastal, inundation, pluvial, storm, hail, hurricane, cyclone, tornado, sea level rise, tsunami, landslide |
|  | Synonym | disaster, catastrophe, climate hazard |
| Model type | Type | catastrophe model, damage model, actuarial model, insurance supply model, econometric model, agent-based model, ABM, machine learning model, machine learning, deep learning |
|  | Data | geospatial model, GIS |
| Insurance | - | insurance, compensation system, compensation arrangement, reinsurance, microinsurance, actuarial |

The final search string has the following form:

TITLE-ABS-KEY ( **flood*** OR **river*** OR **coast*** OR **inundation** OR **pluvial** OR **storm*** OR **hail** OR **hurricane** OR **cyclone** OR **tornado** OR **"sea level rise"** OR **landslide** OR **wildfire** OR **drought** OR climat* ) AND TITLE-ABS-KEY ( **model*** OR **abm** OR **"machine learning"** OR **"deep learning"** OR **gis** OR **"geo information science"** OR **ai** OR **"artificial intelligence"** ) AND TITLE-ABS-KEY ( **insur*** OR **"compensation arrangement"** OR **"compensation system"** OR **reinsur*** OR **microinsurance** OR **actuar*** ) AND ( LIMIT-TO ( LANGUAGE , **"English"** ) ) AND ( LIMIT-TO ( DOCTYPE , **"ar"** ) )

**Supplementary Table 2:**

| **Paper** | **Reason for exclusion** |
| --- | --- |
| Botzen and van den Bergh^1^ | No premium calculated |
| De Koning et al.^2^ | No premium calculated |
| Dietz and Niehörster^3^ | Paper is exclusively about reinsurance |
| Gulcubuk and Gunes^4^ | Not an insurance model |
| Hikmah et al.^5^ | No premium calculated |
| Kokot et al.^6^ | Paper is about index / parametric insurance |
| Li et al.^7^ | Paper is about index / parametric insurance |
| Lyubchich and Gel^8^ | No premium calculated |
| Markandya et al.^9^ | Not an insurance model |
| Mohor and Mendiondo^10^ | Paper is about index / parametric insurance |
| Thistlethwaite^11^ | Not an insurance model |
| Wang et al.^12^ | No premium calculated |
| Wilks and Horowitz^13^ | Not an insurance model |

**Supplementary Table 3: Risk and insurance model types**

| **Paper** | **Risk model type** | **Insurance model type** |
| --- | --- | --- |
| Aerts and Botzen^14^ | Catastrophe | Pricing (supply model) |
| Barreal et al.^15^ | Actuarial | Partial equilibrium |
| Birghila et al.^16^ | Catastrophe | Demand model |
| Boudreault and Ojeda^17^ | Catastrophe | Pricing (supply model) |
| Boudreault et al.^18^ | Catastrophe | Pricing (supply model) |
| Brunette et al.^19^ | Actuarial | Pricing (supply model) |
| Brunette et al.^20^ | Theoretical | Partial equilibrium |
| Crick et al.^21^ | Catastrophe | Agent based |
| De Ruig et al.^22^ | Catastrophe | Agent based |
| De Ruig et al.^23^ | Catastrophe | Agent based |
| Ding et al.^24^ | Catastrophe | Principal agent |
| Dubbelboer et al.^25^ | Catastrophe | Agent based |
| El-Adaway^26^ | Actuarial | Pricing (supply model) |
| Ermolieva et al.^27^ | Catastrophe | Partial equilibrium |
| Guo et al.^28^ | Catastrophe | Game theory |
| Hudson et al.^29^ | Catastrophe | Partial equilibrium |
| Hudson et al.^30^ | Catastrophe | Partial equilibrium |
| Islam et al.^31^ | Actuarial | Demand model |
| Jenkins et al.^32^ | Catastrophe | Agent based |
| Kalfin et al.^33^ | Actuarial | Pricing (supply model) |
| Kesete et al.^34^ | Catastrophe | Game theory |
| Kousky et al.^35^ | Catastrophe | Pricing (supply model) |
| Kunreuther et al.^36^ | Catastrophe | Pricing (supply model) |
| Kunreuther et al.^37^ | Catastrophe | Partial equilibrium |
| Loisel et al.^38^ | Catastrophe | Partial equilibrium |
| Moosakhaani et al.^39^ | Catastrophe | Game theory |
| Peng et al.^40^ | ~~Econometric /~~ Catastrophe | Game theory |
| Perazzini et al.^41^ | Catastrophe | Partial equilibrium |
| Pinheiro and Ribeiro^42^ | Actuarial | Pricing (supply model) |
| Sacchelli et al.^43^ | Catastrophe | Pricing (supply model) |
| Sidi et al.^44^ | Actuarial | Pricing (supply model) |
| Tanaka et al.^45^ | Catastrophe | Agent based |
| Tesselaar et al.^46^ | Catastrophe | Partial equilibrium |
| Tesselaar et al.^47^ | Catastrophe | Partial equilibrium |
| Tesselaar et al.^48^ | Catastrophe | Partial equilibrium |
| Thompson et al.^49^ | Actuarial | Pricing (supply model) |
| Unterberger et al.^50^ | Catastrophe | Partial equilibrium |
| Walker et al.^51^ | Catastrophe | Pricing (supply model) |

**Supplementary Table 4: Risk characteristics of the models**

| **Paper** | **Hazard type** | **Multi-hazard / single-hazard** | **Country/region** | **Climate change scenario(s) inclusion** | **Socioeconomic development scenario(s) inclusion** | **Adaptation inclusion** **(by household/government/business)** |
| --- | --- | --- | --- | --- | --- | --- |
| Aerts and Botzen^14^ | Flooding (coastal and riverine) | Single-hazard | The Netherlands | Yes, four climate change scenarios | Yes, future land-use maps based on two economic growth scenarios | Yes (g) |
| Barreal et al.^15^ | Wildfire | Single-hazard | Spain | No | No | Yes (b) |
| Birghila et al.^16^ | Drought | Single-hazard | Austria | Yes, RCP4.5 | No | Yes (b) |
| Boudreault and Ojeda^17^ | Flooding (riverine) | Single-hazard | Canada | No | No | No |
| Boudreault et al.^18^ | Flooding (riverine) | Single-hazard | Canada | Yes, RCP4.5 and RCP8.5 | No | No |
| Brunette et al.^19^ | Wildfire, wind throw, insect outbreaks | Multi-hazard | Slovakia | No | No | No |
| Brunette et al.^20^ | Damage to forest in general | Single-hazard and multi-hazard | - | No | No | Yes (b) |
| Crick et al.^21^ | Flooding (surface water) | Single-hazard | UK, London | Yes, a baseline and high-emission scenario | No | Yes (g [and developers]) |
| De Ruig et al.^22^ | Flooding (coastal and riverine) | Single-hazard (flooding seen as one type) | United States | Yes, RCP4.5 and RCP8.5 | Yes, SSP2 and SSP5 | Yes (h + g) |
| De Ruig et al.^23^ | Flooding (coastal) | Single-hazard | USA, New York City: Jamaica Bay | Yes, sea level rise | Yes, SSP2 and SSP5 | Yes (h) |
| Ding et al.^24^ | Debris flows | Single-hazard | China, Shengou Basin | No | No | No |
| Dubbelboer et al.^25^ | Flooding (surface water) | Single-hazard | UK, London | Yes, high-emission scenario | No | Yes (h + g) |
| El-Adaway^26^ | Windstorms | Single-hazard | United States, Mississippi | No | No | No |
| Ermolieva et al.^27^ | Flooding (riverine and coastal) | Single-hazard | The Netherlands, Rotterdam | No | No | No |
| Guo et al.^28^ | Hurricanes (flood and wind) | Single-hazard | United States, North Carolina | No | No | Yes (h + g) (government does buyouts and offers subsidies) |
| Hudson et al.^29^ | Flooding (riverine) | Single-hazard | France and Germany | Yes, SRES A1B greenhouse gas emission scenario | Yes, socioeconomic projections at the national level from CIESIN | Yes (h) |
| Hudson et al.^30^ | Flooding (riverine) | Single-hazard | European Union | Yes, SRES A1 scenario | Yes, ensemble mean of SSP scenarios | Yes (h + g) |
| Islam et al.^31^ | Flooding (flash floods) | Single-hazard | Bangladesh | No | No | No |
| Jenkins et al.^32^ | Flooding (surface water) | Single-hazard | UK, London: Camden | Yes, high-emission scenario | No | Yes (h + g) |
| Kalfin et al.^33^ | Natural disasters in general | Single-hazard and multi-hazard | Indonesia | No | No | No |
| Kesete et al.^34^ | Hurricanes | Single-hazard | United States, North Carolina | No | No | No |
| Kousky et al.^35^ | Flooding (coastal) | Single-hazard | United States, New York City | Yes, storm tide projections | No | No |
| Kunreuther et al.^36^ | Hurricanes | Single-hazard | United States, Florida | Yes, six future hurricane scenarios | No | Yes (h) |
| Kunreuther et al.^37^ | Wildfire | Single-hazard | United States, California | No | No | Yes (h) |
| Loisel et al.^38^ | Storm | Single-hazard | Southwest France | No | No | No |
| Moosakhaani et al.^39^ | Flooding (riverine) | Single-hazard | Iran | No | No | No |
| Peng et al.^40^ | Hurricanes | Single-hazard (but it covers both wind and flood damage) | USA, North Carolina | No | No | Yes (h + g) |
| Perazzini et al.^41^ | Earthquakes, flooding (general) | Single- and multi-hazard | Italy | No | No | No |
| Pinheiro and Ribeiro^42^ | Wildfire | Single-hazard | Portugal | No | No | No |
| Sacchelli et al.^43^ | Wildfire and storm | Multi-hazard | Italy | No | No | No |
| Sidi et al.^44^ | Flooding (riverine) | Single-hazard | Indonesia | No | No | No |
| Tanaka et al.^45^ | Flooding (riverine/pluvial) | Single-hazard | Japan | Yes, 2- and 4-degree temperature rise | Yes, income and house prices increase over time; economy grows at a constant rate over time | No |
| Tesselaar et al.^46^ | Flooding (riverine) | Single-hazard | European Union + UK | Yes, RCP4.5 and RCP8.5 and the average of five GCM scenarios | Yes, SSP2 and SSP5 and Winsemius et al.’s^52^ future simulations of built-up area | Yes (h) |
| Tesselaar et al.^47^ | Flooding (riverine) | Single-hazard | European Union + UK | Yes, RCP2.6, RCP4.5, RCP6.0, RCP8.5, and the average of five GCM scenarios | Yes, SSP1, SSP2, SSP3, SSP5, and Winsemius et al.’s^52^ future simulations of built-up area | Yes (h) |
| Tesselaar et al.^48^ | Flooding (riverine) | Single-hazard | European Union + UK | Yes, RCP4.5 (more RCP scenarios in the appendix), and the average of CMIP5 GCM scenarios | Yes, SSP2 (more SSP scenarios in appendix), and Winsemius et al.’s^52^ future simulations of built-up area | Yes (h) |
| Thompson et al.^49^ | Wildfires | Single-hazard | Western USA | No | No | No |
| Unterberger et al.^50^ | Flooding (riverine) | Single-hazard | Austria | Yes, RCP8.5 and 5 GCM scenarios | Yes, SSP5 | Yes (g) |
| Walker et al.^51^ | Cyclones | Single-hazard | Australia | Yes, maximum wind speeds increase by 5% each year over a 90-year period | Yes, vulnerability curve is shifted such that higher wind speeds cause the same level of damage | Yes (h) |

**Supplementary Table 5: Insurance characteristics of the models**

| **Paper** | **Consumer type** | **Insurance markets** | **Competition between insurers modeled** | **Commercial sector insurance** | **Premium setting** | **Decision to insure** |
| --- | --- | --- | --- | --- | --- | --- |
| Aerts and Botzen^14^ | Households | Private and public–private | No | No | Risk-based with a premium loading factor to account for insurance company costs and profit; insurers pay up to a cap, and the government pays the rest. | Households are obliged to take flood insurance. |
| Barreal et al.^15^ | Forestry sector | - | No | Yes | The optimal premium is calculated between the difference in expected damage with and without insurance. | Based on the net present value of forest investments |
| Birghila et al.^16^ | Farmers | One representative insurer | No | Yes | The premium is based on the distortion premium principle, where the loss suffered is multiplied by a distortion risk measure, which leads to higher values for low-probability high-consequence events. The premium also contains a loading factor. The policyholder has a budget of which a proportion is available for the premium (representing a deductible). The government also finances a proportion of the premium. | Only the middle losses are potentially insured; small losses are for the insurance taker, and large losses require outside assistance. How large the proportion of middle losses is is determined via an optimization problem. The optimization problem is based on the loss distribution for the case of only a single loss distribution (non-ambiguous case) or the case of multiple loss distributions (ambiguous case). The policyholder is expected to be risk neutral for small losses and risk averse for large losses. The policyholder has a budget of which a proportion is available for the premium (representing a deductible). |
| Boudreault and Ojeda^17^ | Households | Two competing insurance companies | Yes | No | The insurance company clusters the households based on similar flood risk and bases the premium on the average annual loss per cluster. | Homeowners choose the insurance contract with the lowest premium. |
| Boudreault et al.^18^ | Households | One representative insurer | No | No | A base premium times the relative riskiness and the exposure or a risk-sharing parameter (to spread the risk across all policyholders in the portfolio) instead of the relative riskiness; if this is too low, the flood loss does include a deductible and a limit. The relative riskiness is calculated per region. | NA |
| Brunette et al.^19^ | Forestry sector | One representative insurer | No | Yes | Risk-based + extra premium to insure the forest stand in relation to the total insured area of hectares to reduce the risk of the insurer. | NA |
| Brunette et al.^20^ | Forestry sector | One representative insurer | No | Yes | The insurer is risk neutral, and the price of insurance is given as the unit price of insurance times the compensation amount. The insurance contract includes a deductible that can be chosen by the insured. | Based on a strictly increasing and concave utility function (risk aversion) |
| Crick et al.^21^ | Households | Public market with public reinsurance scheme FloodRe | No | No | Risk-based taking risk-reduction methods into account, a deductible, and a base premium. The impact of the reinsurance scheme FloodRe on the premium is also calculated. | Households are obliged to take flood insurance. |
| De Ruig et al.^22^ | Households | Public insurance program with four different structures | No | No | Based on old national flood insurance program premiums / risk-based with a risk reduction premium discount | Subjective expected utility function and affordability |
| De Ruig et al.^23^ | Households | Public insurance program with four different structures | No | No | Based on old national flood insurance program premiums / risk-based with a risk reduction premium discount | Subjective expected utility function that accounts for bounded rationality in forming risk perceptions and affordability |
| Ding et al.^24^ | Households | One representative insurer | No | No | The premium is chosen by the insurance company alongside the rate of compensation based on an income-maximizing problem that considers the risk degree of debris flow, the total insured assets, the premium, the rate of compensation, and the loss caused by debris flow. | The decision to purchase insurance is based on an expected utility curve that takes into account the risk degree of debris flow, the total assets of the insurant, the loss caused by debris flows, the premium, and the rate of compensation. |
| Dubbelboer et al.^25^ | Households | Public market with public reinsurance scheme FloodRe | No (mentions what could happen if competition was modeled) | No | Risk-based taking risk-reduction methods into account, a deductible, and a base premium. The impact of the reinsurance scheme FloodRe on the premium is also calculated. | Households are obliged to take flood insurance. |
| El-Adaway^26^ | Civil infrastructure developments | One representative insurer | No | No | Based on running a Monte Carlo simulation on bootstrapped historical loss data. | NA |
| Ermolieva et al.^27^ | Households and firms | Insurers, governments, reinsurers, and funds are grouped via a risk reserve. In the case study, only one insurer or catastrophe fund operates per region. | No | Yes | Using quantile-based stochastic optimization under a range of safety constraints across stakeholders to produce optimal risk-based location-specific insurance premiums and coverage. | The decision variables, including insurance coverage, is determined via the optimization of the system. |
| Guo et al.^28^ | Households | A market of multiple insurers, incorporated via a perfect information Cournot–Nash noncooperative game | Yes | No | The premium price is defined as charge per expected dollar loss and varies by region. Hypothetical insurance price levels are first simulated to maximize the insurers’ net profits, and these insurance prices are then implemented in the Cournot–Nash equilibrium model to generate the equilibrium risk-based insurance prices. | Based on a mixed logit model using the insurance premium, the deductible, an indicator of whether the home is located inside or outside the floodplain, the house-to-coastline distance, the number of hurricanes experienced by the homeowner, the homeowner’s income, age, and years since the last hurricane experienced as covariates. There is also an affordability constraint such that the premium cannot exceed the homeowner’s budget expressed as a percentage of the home value. |
| Hudson et al.^29^ | Households | Six market structures, ranging from public to private | Yes (in appendix) | No | Risk-based and depending on the number of households in a region, the premium includes a loading factor and a deductible; there is an incentive in the premium for DRR methods: The premium gets lower via the effectiveness ratio of the DRR method. | Subjective expected utility that accounts for affordability |
| Hudson et al.^30^ | Households | A public–private flood insurance scheme | No | No | Various insurance premium rules, including risk-based, risk-based with a risk reduction premium discount, capped risk-based premium, and solidarity premium. | Subjective expected utility that accounts for affordability |
| Islam et al.^31^ | Crop insurance | Only an insurance premium is calculated. | No | Yes | Damage based for different coverage levels and interest rates | Binary logistic regression approach to elicit the willingness to adopt insurance |
| Jenkins et al.^32^ | Households | Public market with public reinsurance scheme FloodRe | No (mentions what could happen if competition was modeled) | No | Expected annual loss of the insurers minus the excesses and base flood insurance premium; the remaining loss is spread across the households based on risk. There is an option for reinsurance and risk reduction measures. | Households are obliged to take flood insurance. |
| Kalfin et al.^33^ | Households | No explicit interaction between insurers | Yes (model that the paper expands on mentions competition) | No | Risk-based with a system that taxes the low-risk areas to provide a subsidy to the high-risk areas | NA |
| Kesete et al.^34^ | Households | One representative insurer | No | No | Premiums are set via a Stackelberg leader–follower game, where the insurer determines the price of the premium, and the homeowner decides whether or not to purchase insurance based on a utility function. One reinsurer provides reinsurance at a specified price, and the government can set constraints to both the insurer and the homeowners. The premium gets determined via stochastic optimization of this system, where the insurer wants to optimize its profit, avoid insolvency, and maintain sufficient yearly profitability. The premium includes a deductible and loading factor for the insurer’s administration costs and profit margin. The premium should be greater than a certain value. | Based on an expected utility function where the homeowners are expected to be risk-averse and have a maximum budget for insurance based on a percentage of the home value. |
| Kousky et al.^35^ | Households | Public insurance program | No | No | Property specific premium calculator | NA |
| Kunreuther et al.^36^ | Households | Two different insurance markets: a hard (with capital scarcity and high reinsurance prices) and a soft one (with capital abundance and low reinsurance prices). One representative insurer. | No | No | Risk-based with a loading factor to cover additional cost (this loading factor is not used in the case study). The premium also considers a risk aversion parameter. The premium is calculated for both the insurer and reinsurer. | NA |
| Kunreuther et al.^37^ | Households | Multiple insurers | No | No | In the original simulation, premiums are actuarially fair plus a loading factor but it is noted that in reality premiums are probably set to reflect the risk but also to generate a profit. | Homeowners decide whether or not to take out insurance by taking into account potential mitigation benefits. |
| Loisel et al.^38^ | Forestry sector | One representative insurer | No | Yes | The insurance premium is a decision variable in the model and can be set by the insurer. The insurer sets the insurance premium by making sure it is more than the future losses multiplied by a discount rate connected to the cutting age of the trees. The premium also includes a loading factor. The cutting age of the trees is determined by the forest owner and determines the forest land expected value (Faustmann value). The insurer sets an upper premium, the premium that is used when the forest owner decides to fully insure their forest. | The decision to insure is given by a decision variable that takes values between 0 and 1, where 1 means full insurance, and 0 means no insurance. The decision to insure depends on the Faustmann value, which stands for the forest land expected value. |
| Moosakhaani et al.^39^ | Households and government | Three insurers that all have an insurance plan for property owners and an insurance plan for the government | Yes | No | The premium includes a deductible and a fixed number for each loss to cover other costs. | The property owners and the government decide to buy insurance based on their payoff functions. The decision is determined via the Nash equilibrium in a game-theoretic approach where the insurers, property owners, and the government play a role. Government compensation also plays a role, but keep in mind that in this paper, the insurers also offer insurance to the government. |
| Peng et al.^40^ | Households | One representative insurer | No | No | The premium is based on the expected value of the loss per building type considering coverage of the full home value with a specified deductible. The premium also contains two loading factors: one for the administrative costs, and one for the profit margin per risk region. The insurer is expected to maximize its profit. In this scheme, retrofitting decreases the expected loss and, thereby, the premium charged. There is a minimum premium threshold. | The model is run for five configurations, either allowing or disallowing retrofitting with or without a government subsidy and mandatory or voluntary insurance. Under voluntary insurance, the decision to insure is determined via a Stackelberg game between the insurer and the homeowners. The insurer determines the premiums of various policies and decides how much reinsurance to purchase. The homeowner then decides what policy and/or what retrofit options they want to purchase. The decision-making of the homeowners is ultimately decided via utility maximization. Each homeowner has a maximum budget for homeowner insurance equal to a specified percentage of the total home value; this percentage varies per risk region. |
| Perazzini et al.^41^ | Households | Private and public–private insurance | Yes (but only mentions) | No | Risk-based plus profit loading, where the risk-based premium should be in the premium the households are willing to pay, and the profit should be adequate. | Willingness to pay calculated using a utility function expecting the homeowners to be rational and risk-averse. |
| Pinheiro and Ribeiro^42^ | Forestry sector | One representative insurer | No | Yes | The premium is based on the financial risk of the insurer which is based on the expected burned area and the forest stand expected value. | The maximum and minimum premium a forestry farmer is willing to pay is based on the financial risk for the forestry farmer, which is based on the frequency of fire and the farm size. |
| Sacchelli et al.^43^ | Forestry sector | No explicit interaction between insurers | No | Yes | A base premium based on the expected annual loss with a risk premium to allow insurance companies to prepare for extreme years, variable management costs, and fixed insurance company costs. | NA |
| Sidi et al.^44^ | Buildings | No explicit interaction between insurers | No | No | The premium is calculated as random variable via different methods, via the Esscher principle, based on the proportional hazards approach, Wang’s models, Swiss model, and Dutch model. | NA |
| Tanaka et al.^45^ | Households | No explicit interaction between insurers | No | No | The insurance premium is given per house type per period based on the expected annual flood damage ratio. The insurance rate is multiplied by a markup rate that reflects risk aversion. The flood risk is expected to be perfectly reflected in the insurance premium with the markup rate. | The ratio of households that anticipate flood risk is given; these households will buy insurance. Households take full insurance. |
| Tesselaar et al.^46^ | Households | Flood insurance systems that involve a reinsurer: a voluntary system and a semi-voluntary system | Yes | No | The premium is risk-based and includes a deductible of 15% of the loss. The insurer also increases the premium due to the uncertainty of damage by multiplying a risk aversion coefficient with the volatility of damage; 99.8% of damages are considered insurable. The insurer cannot charge a profit loading factor but does charge a cost loading factor. Based on market-structure, there might also be mandatory insurance. | The decision to insure is based on a subjective expected utility function. Households can receive a discount on the premium by implementing disaster reduction measures. The premium should be equal or smaller than the poverty-adjusted disposable income. |
| Tesselaar et al.^47^ | Households | Six stylized insurance market structures, ranging from full mandatory to full voluntary and from public to private. Four market forms are stylized to European insurance markets, and two are hypothetical. | Yes | No | The premium is risk-based and includes a deductible of 15% of the loss. The insurer also increases the premium due to the uncertainty of damage by multiplying a risk aversion coefficient with the volatility of damage; 99.8% of damages are considered insurable. The insurer cannot charge a profit loading factor but does charge a cost loading factor. Based on market-structure, there might also be mandatory insurance. | The decision to insure is based on a subjective expected utility function. Households can obtain a discount on the premium by implementing disaster reduction measures. The premium should be equal or smaller than the poverty-adjusted disposable income. |
| Tesselaar et al.^48^ | Households | Four market structures, ranging from full mandatory to full voluntary, with either a risk-based or a non-risk-based premium | Yes | No | Based on the market structure. In the voluntary system, the premiums are risk-based, 99.8% of damages are considered insurable, a deductible is set as 15% of the loss, there is a risk aversion parameter that increases the premium, and there is no profit loading factor, but there is a cost loading factor. Households receive a premium discount if they implement risk reduction measures; the premium also contains a reinsurance premium that is calculated in a similar way as the insurance premium but does contain a profit loading factor of 50% of the underwritten risk. In the semi-voluntary system, the premiums are still risk-based, but many households are mandated to take insurance. In the solidarity system, the premiums are insensitive to risk, and all households are mandated to take insurance. In the public–private partnership, the premiums are risk-sensitive up to a certain point; insurance uptake is mandatory for mortgage holders. | If the premium does not cause the household to fall under the poverty line, the decision to purchase insurance is based on maximizing an expected utility curve, which is also dependent on whether the household anticipated government aid after a flood. |
| Thompson et al.^49^ | Annual wildfire suppression costs | NA | No | NA | The paper couples a wildfire simulation model and a suppression cost model to estimate probability distributions for the suppression costs. The suppression cost of a fire is comparable to an insurance premium. The total suppression costs for a national forest is defined as the sum of all suppression expenditures per escaped large wildfire. The expected value of the suppression cost is calculated by multiplying the expected value of the number of escaped large wildfires in a given fire season by the expected value of the suppression costs per large fire. The variance of the total suppression costs is defined as the expected value of the number of escaped large wildfires times the variance of the suppression costs per large fire plus the expected value of the suppression costs per large fire squared times the variance of the number of escaped large wildfires. | NA |
| Unterberger et al.^50^ | Public infrastructure | Insurance is organized in three market forms: one where there is just a disaster fund, one where there is risk transfer to a private insurer, and one where there is a public–private insurance mechanism. The insurance sector is simplified to one representative insurer. | No | No | The premiums are based on the expected annual damage with a surcharge that represents the volatility in annual losses, which reflects the risk aversion of reinsurers. On top of this premium, there is a further surcharge that is used to cover administrative costs and generate a profit. The premiums are calculated for both federal and regional governments and include a deductible of 15% of the loss suffered. | There is no decision to take up insurance, but there is a decision to employ dry flood-proofing, which is calculated via the net-present value of the reduction in premiums over 20 years (which is considered as the lifespan of the dry flood-proofing measure). |
| Walker et al.^51^ | Constructed assets | There is no explicit interaction between insurers; the model works with one hypothetical insurer. | No | Yes | Premiums are calculated based on the average annual risk of loss multiplied by a risk loading factor that reflects the administrative costs and a term that increases the nonlinearity, essentially reflecting the volatility for the insurer. | NA |

# References

1. Botzen, W. J. W., & Van Den Bergh, J. C. J. M. (2012). Monetary valuation of insurance against flood risk under climate change. *International Economic Review*, *53*(3), 1005–1026. https://doi.org/10.1111/j.1468-2354.2012.00709.x

2. De Koning, K., Filatova, T., & Bin, O. (2019). Capitalization of Flood Insurance and Risk Perceptions in Housing Prices: An Empirical Agent-Based Model Approach. *Southern Economic Journal*, *85*(4), 1159–1179. https://doi.org/10.1002/soej.12328

3. Dietz, S., & Niehörster, F. (2021). Pricing ambiguity in catastrophe risk insurance. *GENEVA Risk and Insurance Review*, *46*(2), 112–132. https://doi.org/10.1057/s10713-020-00051-2

4. Gulcubuk, B., & Gunes, E. (2010). Applicable agricultural insurance models at the rural area: A case study from Turkey. *Scientific Research and Essays*, *5*(9), 837–844. http://www.academicjournals.org/SRE

5. Hikmah, Y., Yuristamanda, V., Hikmah, I. R., & Safitri, K. A. (2022). Probit Modeling of Indonesian Economic and Social Factors to The Interest in Purchasing Flood-Impacted Insurance Products. *International Journal of Industrial Engineering & Production Research*, *33*(2), 1–12. https://doi.org/10.22068/ijiepr.33.2.3

6. Kokot, Ž., Marković, T., Ivanović, S., & Meseldžija, M. (2020). Whole-farm revenue protection as a factor of economic stability in crop production. *Sustainability (Switzerland)*, *12*(16), 205–220. https://doi.org/10.3390/SU12166349

7. Li, L., Liu, Z., Chen, J. Y., Wu, Y. C., & Li, H. (2022). Enhanced Agriculture Insurance with Climate Forecast. *Sustainability (Switzerland)*, *14*(17). https://doi.org/10.3390/su141710617

8. Lyubchich, V., & Gel, Y. R. (2016). Can we weather proof our insurance? *Environmetrics*, *28*(2). https://doi.org/10.1002/env.2433

9. Markandya, A., De Cian, E., Drouet, L., Polanco-Martínez, J. M., & Bosello, F. (2019). Building Risk into the Mitigation/Adaptation Decisions simulated by Integrated Assessment Models. *Environmental and Resource Economics*, *74*(4), 1687–1721. https://doi.org/10.1007/s10640-019-00384-1

10. Mohor, G. S., & Mendiondo, E. M. (2017). Economic indicators of hydrologic drought insurance under water demand and climate change scenarios in a Brazilian context. *Ecological Economics*, *140*, 66–78. https://doi.org/10.1016/j.ecolecon.2017.04.014

11. Thistlethwaite, J. (2017). The Emergence of Flood Insurance in Canada: Navigating Institutional Uncertainty. *Risk Analysis*, *37*(4), 744–755. https://doi.org/10.1111/risa.12659

12. Wang, D., Davidson, R. A., Trainor, J. E., Nozick, L. K., & Kruse, J. (2017). Homeowner purchase of insurance for hurricane-induced wind and flood damage. *Natural Hazards*, *88*(1), 221–245. https://doi.org/10.1007/s11069-017-2863-x

13. Wilks, D. S., & Horowitz, K. A. (2014). A novel financial market for mitigating hurricane risk. Part I: Market structure and model results. *Weather, Climate, and Society*, *6*(3), 307–317. https://doi.org/10.1175/WCAS-D-13-00032.1

14. Aerts, J. C. J. H., & Botzen, W. J. W. (2011). Climate change impacts on pricing long-term flood insurance: A comprehensive study for the Netherlands. *Global Environmental Change*, *21*(3), 1045–1060. https://doi.org/10.1016/j.gloenvcha.2011.04.005

15. Barreal, J., Loureiro, M. L., & Picos, J. (2014). On insurance as a tool for securing forest restoration after wildfires. *Forest Policy and Economics*, *42*, 15–23. https://doi.org/10.1016/j.forpol.2014.02.001

16. Birghila, C., Pflug, G. C., & Hochrainer-Stigler, S. (2022). Risk-layering and optimal insurance uptake under ambiguity: With an application to farmers exposed to drought risk in Austria. *Risk Analysis*, *42*(12), 2639–2655. https://doi.org/10.1111/risa.13884

17. Boudreault, M., & Ojeda, A. (2022). Ratemaking territories and adverse selection for flood insurance. *Insurance: Mathematics and Economics*, *107*, 349–360. https://doi.org/10.1016/j.insmatheco.2022.09.005

18. Boudreault, M., Grenier, P., Pigeon, M., Potvin, J. M., & Turcotte, R. (2020). Pricing Flood Insurance with a Hierarchical Physics-Based Model. *North American Actuarial Journal*, *24*(2), 251–274. https://doi.org/10.1080/10920277.2019.1667830

19. Brunette, M., Holecy, J., Sedliak, M., Tucek, J., & Hanewinkel, M. (2015). An actuarial model of forest insurance against multiple natural hazards in fir (Abies Alba Mill.) stands in Slovakia. *Forest Policy and Economics*, *55*, 46–57. https://doi.org/10.1016/j.forpol.2015.03.001

20. Brunette, M., Couture, S., & Pannequin, F. (2017). Is forest insurance a relevant vector to induce adaptation efforts to climate change? *Annals of Forest Science*, *74*(2). https://doi.org/10.1007/s13595-017-0639-9

21. Crick, F., Jenkins, K., & Surminski, S. (2018). Strengthening insurance partnerships in the face of climate change – Insights from an agent-based model of flood insurance in the UK. *Science of the Total Environment*, *636*, 192–204. https://doi.org/10.1016/j.scitotenv.2018.04.239

22. De Ruig, L. T., Haer, T., de Moel, H., Brody, S. D., Botzen, W. J. W., Czajkowski, J., & Aerts, J. C. J. H. (2022). How the USA can benefit from risk-based premiums combined with flood protection. *Nature Climate Change*, *12*(11), 995–998. https://doi.org/10.1038/s41558-022-01501-7

23. De Ruig, L. T., Haer, T., de Moel, H., Orton, P., Botzen, W. J. W., & Aerts, J. C. J. H. (2023). An agent-based model for evaluating reforms of the National Flood Insurance Program: A benchmarked model applied to Jamaica Bay, NYC. *Risk Analysis*, *43*(2), 405–422. https://doi.org/10.1111/risa.13905

24. Ding, M., Wei, F., & Hu, K. (2012). Property insurance against debris-flow disasters based on risk assessment and the principal-agent theory. *Natural Hazards*, *60*(3), 801–817. https://doi.org/10.1007/s11069-011-9897-2

25. Dubbelboer, J., Nikolic, I., Jenkins, K., & Hall, J. (2017). An Agent-Based Model of Flood Risk and Insurance. *Journal of Artificial Societies and Social Simulation*, 20(1). https://doi.org/10.18564/jasss.3135

26. El-Adaway, I. H. (2012). Insurance Pricing for Windstorm-Susceptible Developments: Bootstrapping Approach. *Journal of Management in Engineering*, *28*(2), 96–103. https://doi.org/10.1061/(asce)me.1943-5479.0000088

27. Ermolieva, T., Filatova, T., Ermoliev, Y., Obersteiner, M., de Bruijn, K. M., & Jeuken, A. (2017). Flood Catastrophe Model for Designing Optimal Flood Insurance Program: Estimating Location-Specific Premiums in the Netherlands. *Risk Analysis*, *37*(1), 82–98. https://doi.org/10.1111/risa.12589

28. Guo, C., Nozick, L., Kruse, J., Millea, M., Davidson, R., & Trainor, J. (2022). Dynamic modeling of public and private decision-making for hurricane risk management including insurance, acquisition, and mitigation policy. *Risk Management and Insurance Review*, *25*(2), 173–199. https://doi.org/10.1111/rmir.12215

29. Hudson, P., Botzen, W. J. W., Feyen, L., & Aerts, J. C. J. H. (2016). Incentivising flood risk adaptation through risk based insurance premiums: Trade-offs between affordability and risk reduction. *Ecological Economics*, *125*, 1–13. https://doi.org/10.1016/j.ecolecon.2016.01.015

30. Hudson, P., Botzen, W. J. W., & Aerts, J. C. J. H. (2019). Flood insurance arrangements in the European Union for future flood risk under climate and socioeconomic change. *Global Environmental Change*, *58*. https://doi.org/10.1016/j.gloenvcha.2019.101966

31. Islam, M. M., Matsushita, S., Noguchi, R., & Ahamed, T. (2022). A damage-based crop insurance system for flash flooding: a satellite remote sensing and econometric approach. *Asia-Pacific Journal of Regional Science*, *6*(1), 47–89. https://doi.org/10.1007/s41685-021-00220-9

32. Jenkins, K., Surminski, S., Hall, J., & Crick, F. (2017). Assessing surface water flood risk and management strategies under future climate change: Insights from an Agent-Based Model. *Science of the Total Environment*, *595*, 159–168. https://doi.org/10.1016/j.scitotenv.2017.03.242

33. Kalfin, Sukono, Supian, S., & Mamat, M. (2022). Insurance Premium Determination Model and Innovation for Economic Recovery Due to Natural Disasters in Indonesia. *Computation*, *10*(10). https://doi.org/10.3390/computation10100174

34. Kesete, Y., Peng, J., Gao, Y., Shan, X., Davidson, R. A., Nozick, L. K., & Kruse, J. (2014). Modeling Insurer-Homeowner Interactions in Managing Natural Disaster Risk. *Risk Analysis*, *34*(6), 1040–1055. https://doi.org/10.1111/risa.12227

35. Kousky, C., Kunreuther, H., Xian, S., & Lin, N. (2021). Adapting our Flood Risk Policies to Changing Conditions. *Risk Analysis*, *41*(10), 1739–1743. https://doi.org/10.1111/risa.13692

36. Kunreuther, H., Michel-Kerjan, E., & Ranger, N. (2013). Insuring future climate catastrophes. *Climatic Change*, *118*(2), 339–354. https://doi.org/10.1007/s10584-012-0625-z

37. Kunreuther, H., Demidov, A., Pauly, M., Turcic, M., & Wilson, M. (2023). Externalities in the wildland–urban interface: Private decisions, collective action, and results from wildfire simulation models for California. *Risk Analysis*, *43*(5), 886–895. https://doi.org/10.1111/risa.14135

38. Loisel, P., Brunette, M., & Couture, S. (2020). Insurance and Forest Rotation Decisions Under Storm Risk. *Environmental and Resource Economics*, *76*(2–3), 347–367. https://doi.org/10.1007/s10640-020-00429-w

39. Moosakhaani, M., Salimi, L., Sadatipour, M. T., Niksokhan, M. H., & Rabbani, M. (2022). Game theoretic approach for flood risk management considering a financial model. *Environmental Engineering Research*, *27*(6). https://doi.org/10.4491/eer.2021.368

40. Peng, J., Shan, X. G., Gao, Y., Kesete, Y., Davidson, R. A., Nozick, L. K., & Kruse, J. (2014). Modeling the integrated roles of insurance and retrofit in managing natural disaster risk: a multi-stakeholder perspective. *Natural Hazards*, *74*(2), 1043–1068. https://doi.org/10.1007/s11069-014-1231-3

41. Perazzini, S., Gnecco, G., & Pammolli, F. (2022). A Public–Private Insurance Model for Disaster Risk Management: An Application to Italy. *Italian Economic Journal*. https://doi.org/10.1007/s40797-022-00210-6

42. Pinheiro, A., & Ribeiro, N. (2013). Forest property insurance: an application to Portuguese woodlands. *International Journal Of Sustainable Society*, *5*(3), 284. https://doi.org/10.1504/ijssoc.2013.054716

43. Sacchelli, S., Cipollaro, M., & Fabbrizzi, S. (2018). A GIS-based model for multiscale forest insurance analysis: The Italian case study. *Forest Policy and Economics*, *92*, 106–118. https://doi.org/10.1016/j.forpol.2018.04.011

44. Sidi, P., Mamat, M., Sukono, & Supian, S. (2017). Evaluation Model for Risk Insurance Premiums of Building Damage Caused by Flood: Case Study in Citarum Watershed, Southern Bandung, Indonesia. *Journal of Engineering and Applied Sciences*, *12*(17), 4420–4425.

45. Tanaka, T., Yokomatsu, M., Ashino, M., & Ichikawa, Y. (2022). Novel framework for assessing long-term flood risk management pathways focusing on river channel improvement and amenity policies. *Journal of Flood Risk Management*, *15*(3). https://doi.org/10.1111/jfr3.12804

46. Tesselaar, M., Botzen, W. J. W., & Aerts, J. C. J. H. (2020). Impacts of climate change and remote natural catastrophes on EU flood insurance markets: An analysis of soft and hard reinsurance markets for flood coverage. *Atmosphere*, *11*(2). https://doi.org/10.3390/atmos11020146

47. Tesselaar, M., Wouter Botzen, W. J., Haer, T., Hudson, P., Tiggeloven, T., & Aerts, J. C. J. H. (2020). Regional inequalities in flood insurance affordability and uptake under climate change. *Sustainability (Switzerland)*, *12*(20), 1–30. https://doi.org/10.3390/su12208734

48. Tesselaar, M., Botzen, W. J. W., Robinson, P. J., Aerts, J. C. J. H., & Zhou, F. (2022). Charity hazard and the flood insurance protection gap: An EU scale assessment under climate change. *Ecological Economics*, *193*. https://doi.org/10.1016/j.ecolecon.2021.107289

49. Thompson, M. P., Haas, J. R., Finney, M. A., Calkin, D. E., Hand, M. S., Browne, M. J., Halek, M., Short, K. C., & Grenfell, I. C. (2015). Development and application of a probabilistic method for wildfire suppression cost modeling. *Forest Policy and Economics*, *50*, 249–258. https://doi.org/10.1016/j.forpol.2014.10.001

50. Unterberger, C., Hudson, P., Botzen, W. J. W., Schroeer, K., & Steininger, K. W. (2019). Future Public Sector Flood Risk and Risk Sharing Arrangements: An Assessment for Austria. *Ecological Economics*, *156*, 153–163. https://doi.org/10.1016/j.ecolecon.2018.09.019

51. Walker, G. R., Mason, M. S., Crompton, R. P., & Musulin, R. T. (2016). Application of insurance modelling tools to climate change adaptation decision-making relating to the built environment. *Structure and Infrastructure Engineering*, *12*(4), 450–462. https://doi.org/10.1080/15732479.2015.1020498

52. Winsemius, H. C., Aerts, J. C. J. H., Van Beek, L. P. H., Bierkens, M. F. P., Bouwman, A., Jongman, B., Kwadijk, J. C. J., Ligtvoet, W., Lucas, P. L., Van Vuuren, D. P., & Ward, P. J. (2016). Global drivers of future river flood risk. *Nature Climate Change*, *6*(4), 381–385. https://doi.org/10.1038/nclimate2893
